# Direct sequencing of measles virus complete genomes in the midst of a large-scale outbreak

**DOI:** 10.1371/journal.pone.0255663

**Published:** 2021-09-10

**Authors:** Efrat Bucris, Victoria Indenbaum, Roberto Azar, Oran Erster, Eric Haas, Ella Mendelson, Neta S. Zuckerman

**Affiliations:** 1 Central Virology Laboratory, Ministry of Health, Chaim Sheba Medical Center, Tel-Hashomer, Ramat-Gan, Israel; 2 Division of Epidemiology, Israel Ministry of Health, Jerusalem, Isreal; 3 Faculty of Health Sciences, Ben Gurion University, Be’er Sheva, Israel; 4 School of Public Health, Tel Aviv University, Tel Aviv-Yafo, Israel; University of Helsinki: Helsingin Yliopisto, FINLAND

## Abstract

Measles outbreaks escalated globally despite worldwide elimination efforts. Molecular epidemiological investigations utilizing partial measles virus (MeV) genomes are challenged by reduction in global genotypes and low evolutionary rates. Greater resolution was reached using MeV complete genomes, however time and costs limit the application to numerous samples. We developed an approach to unbiasedly sequence complete MeV genomes directly from patient urine samples. Samples were enriched for MeV using filtration or nucleases and the minimal number of sequence reads to allocate per sample based on its MeV content was assessed using in-silico reduction of sequencing depth. Application of limited-resource sequencing to treated MeV-positive samples demonstrated that 1–5 million sequences for samples with high/medium MeV quantities and 10–15 million sequences for samples with lower MeV quantities are sufficient to obtain >98% MeV genome coverage and over X50 average depth. This approach enables real-time high-resolution molecular epidemiological investigations of large-scale MeV outbreaks.

## Introduction

Measles outbreaks, caused by the measles virus (MeV), have escalated globally with a 300% increase in 2019 compared to the previous year [1], including numerous importation events into non-endemic countries. Standard questionnaire-based epidemiological investigations struggle to reliably differentiate between transmission chains during such large-scale outbreaks. Likewise, molecular characterizations based on partial MeV genomes lack the necessary resolution to describe individual transmission events in cases of reduced genotype diversity [2], low number of linked cases, or where MeV is repeatedly imported from an ongoing large outbreak [3]. For example, laboratories perform measles genotyping utilizing standard amplicon sequencing of the N450 target, which varies by no more than one nucleotide per sequence. However, it is difficult to decipher transmission chains and perform molecular epidemiology investigations based on such partial sequences due to limited resolution [3]. Additionally, a study comparing between different measles genes compared to whole genomes concluded that the whole genome provides higher resolution [4].

MeV complete genomes were shown to enhance the resolution of molecular epidemiologic analyses in smaller-scale outbreaks. However, the MeV-enrichment and sequencing approaches used involved laborious and time-consuming techniques such as targeted sequencing using specific primers [2, 4] and isolates [5] or Sanger sequencing of overlapping amplicons [4]. Next generation sequencing (NGS) of MeV complete genomes, applied directly to clinical samples, has the potential to expeditiously and unbiasedly characterize the large volume of samples necessary to analyze during large-scale outbreaks. Direct sequencing of viruses from patient samples has been applied for viral detection and genotyping [9], where viral discovery via sequencing was shown to be enhanced by viral-enrichment strategies [6, 7]. Indeed, sample preparation prior to sequencing, most commonly including filtration and nuclease digestion that rely on the stability of viral capsids and their small size, have been demonstrated to remove background host and bacterial genomes that hamper reliable recovery of viral genomes from clinical samples [8, 9].

Herein we developed an approach to sequence complete MeV genomes directly from patient clinical samples. To reasonably apply the approach to sequence numerous samples during a large-scale outbreak, we minimized NGS costs per sample by limiting the number of allocated sequence reads based on MeV content in the sample. This approach may ultimately promote high-resolution characterization of large-scale measles outbreaks at the time they occur.

## Materials and methods

### Study samples

The Israeli measles outbreak Israel included >4000 cases. Real-time RT-PCR of MeV content in >1300 outbreak specimen showed a median of Ct = 23, with ~30% of samples having high MeV content (Ct< = 20). For this study, 41 MeV-positive urine samples were chosen from leftovers of clinical samples, kept at 4°C.

#### Ethics approval statement

The study was conducted according to the guidelines of the Declaration of Helsinki, and approved by the Institutional Review Board of the Sheba Medical Center institutional review board (5800-18-SMC). Patient consent was waived because the study used remains of clinical samples and the analysis used anonymous clinical data.

### Pre-treatment and nucleic acid extraction

Samples were freeze-thawed three times to rupture cell membranes, centrifuged at high speed for 20 minutes and treated with a single/combination of treatments: OmniCleave endonuclease, which cleaves free DNA/RNA (Epicenter, Madison, USA) and 0.22μm and/or 0.45μm filters. 200μl of each urine sample was incubated with OmniCleave (250U) in the presence of 2.5mM MgCl2 for 1 hour at 37˚C. Filtering was performed by syringe-driven 13mm PVDF 0.22 and/or 0.45um filters (Merck Millipore, USA). Total nucleic acids from 200μl were extracted from treated and untreated MeV-positive urine sample using NucliSENS easyMAG (bioMérieux Inc, Durham USA), elution performed in 55μl.

### Real-time RT-PCR

MeV-RNA content in each sample before and after treatment was measured by real-time RT-PCR. Negative samples were assigned cycle threshold (Ct) of 50 for clarity, as there are maximum 50 cycles in the assay. Following nucleic acid extraction, samples were tested via ABI7500 RT-PCR system (Thermo Fisher Scientific) for MeV, human cells (RNaseP) and bacteria (16S rRNA) using specific primers and probes. Further details in the S1 File.

### Library preparation and sequencing

RNA libraries were prepared with SMARTer Stranded RNA-Seq kit (Takara, USA). Briefly, first cDNA strand was synthesized, then purified using AMPureXP magnetic beads (Beckman Coulter, USA). RNA libraries were obtained using DNA polymerases and library amplification was done by PCR. Libraries were purified with AMPureXP beads (Beckman Coulter, USA). Library concentration was measured by Qubit dsDNA HS Assay Kit (Thermo Fisher Scientific). Library validation and mean fragment size was determined by TapeStation4200 (Agilent, USA) with DNA High-Sensitivity D1000 (Agilent, USA). Library mean fragment size (~300bp) and concentration molarity were calculated and each library was diluted to 4nM. Libraries were pooled, denatured and diluted to 15pM and sequenced on HiSeq2500 (Illumina, USA), 2X100bp. To allocate the number of reads per sample, quantities from each sample were taken during pooling of libraries based on the desired number of allocated reads. E.g., for a pool of samples to be allocated 1M, 5M and 10M reads—a volume of 1μl, 5μl and 10μl from each sample, respectively, was pooled.

### Bioinformatics analyses

Quality control was applied to fastq files with FastQC (www.bioinformatics.babraham.ac.uk/projects/fastqc/) and MultiQC [10]. MeV reference genome (KT732261.1) was indexed using BWA [11]. BWAmem was used to map fastq files to the indexed reference [11]. SAMtools suite [12, 13] was used to remove duplicates, filter unmapped reads and sort and index BAM files. Coverage and depth of sequencing were calculated from sorted BAM files using a custom script. Consensus sequences were constructed using SAMtools mpileup and bcf tools [14]. Fastq files were subsampled in the range of 250K-15M total reads using seqtk with sample parameter (https://github.com/lh3/seqtk, V1.0). The bioinformatics pipleline described above was applied to the subsampled fastq files. All relevant sequences are available in GenBank (accession numbers: MZ712065- MZ712087).

## Results

### MeV-enrichment reduces background genomes

**Fig 1:**
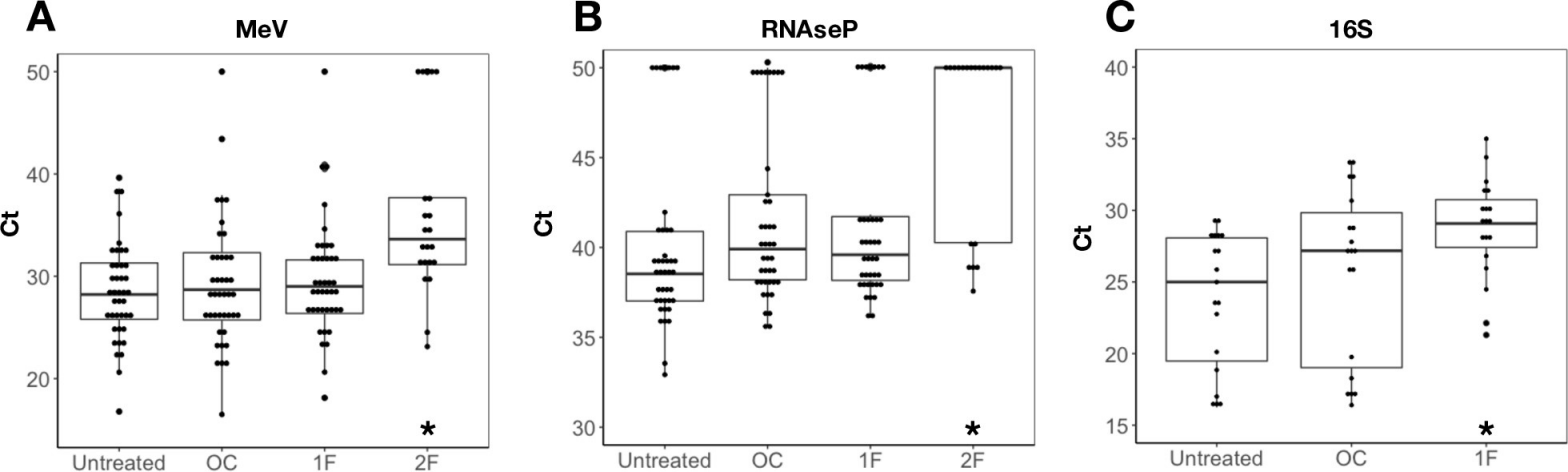
Impact of virus enrichment treatments on MeV, human and bacterial content in clinical samples. 41 MeV-positive urine samples were treated with OmniCleave (OC), 0.45μm filter (1F) or 0.45μm filter followed by 0.22μm filter (2F). The content of MeV, human cells (RNAseP marker) and bacteria (16S marker) was tested prior (untreated) and following each treatment via real-time RT-PCR with specific primers and probes. (a) MeV Ct values for all samples (n = 41). (b) RNAseP Ct values for all samples (n = 41). (c) 16S Ct values for samples for samples with high bacterial content (16S Ct<30) prior to treatment (n = 19). 16S results for all samples (n = 41) is presented in S1 File and Fig 1. Asterisks represent significant differences compared to “untreated” (T-test, p-values<0.05).

MeV-positive urine samples (median MeV Ct = 28) were treated with either nuclease, different sized filters (0.22 and/or 0.45μm) or their combination. The content of MeV, human cells and bacteria were measured via real-time RT-PCR prior to and following each treatment to examine its impact. The nuclease and 0.45μm filter treatments did not alter MeV quantities significantly in any of the samples. Application of two filters, 0.45μm followed by 0.22μm, significantly decreased MeV content in the sample, elevating MeV Ct values to a median of Ct = 36.2 (Fig 1A, (S1 Table in S1 File)). Thus, subsequent experiments included the 0.45μm filter only and compare its effectiveness to that of the nuclease treatment, both of which retained MeV in the samples.

The content of RNAseP, a marker for human cells, was low in most untreated samples, as expected in urine samples. Enrichment treatments further decreased RNAseP content, with median Ct values increasing from 38.5 in untreated samples to 39.9 and 39.5 following endonuclease and 0.45μm filter, respectively. The two-filter treatment significantly decreased RNAseP, similar to its impact on MeV (Fig 1B and (S1 Table in S1 File)).

The content of 16S, a marker for bacteria, was reduced by nuclease and 0.45μm filter treatments, more significantly in samples with high bacterial content prior to treatment. In these samples, the filter treatment more effectively reduced bacterial content compared to nuclease treatment, with 16S median Ct values increasing from 25 in untreated samples to 29 and 27 following filter and nuclease treatments, respectively (Fig 1C, (S1 Fig in S1 File)).

These results demonstrate that both nuclease and 0.45μm filter treatments purify urine samples from human and bacterial nucleic acids by orders of magnitude.

### MeV complete genomes sequenced directly from clinical samples

**Fig 2:**
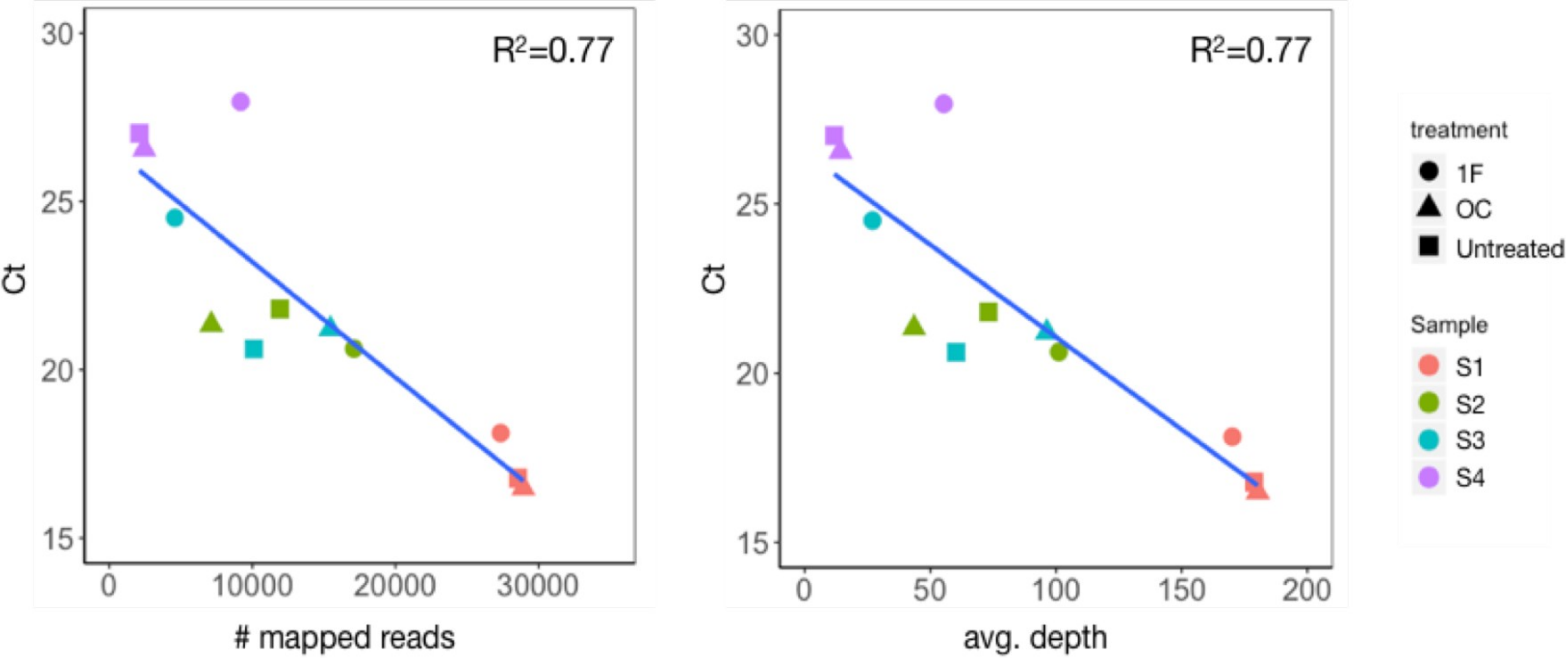
Correlation of Ct values and sequencing parameters. Significant correlations observed between (a) MeV Ct values and (b) average sequencing depth.

To examine the impact of the different enrichment treatments on MeV sequencing, 4 sets of MeV samples prior to and following treatments, with high (Ct = 16–18, sample S1), median (Ct = 20–24, samples S2 and S3) and low (Ct>26, sample S4) MeV content, were sequenced. The total sequences per sample was 20–45 million (M) and the number of sequences that mapped to MeV were 4,500–28,000 (0.01–0.13% of total number of reads, Table 1) and correlated with MeV Ct values (Fig 2A). 10/12 samples had full MeV genome coverage (100%) and the remaining two had >99% coverage and contained lower MeV quantities (Ct = 27–28). Average sequencing depth was 11–180 and correlated with MeV Ct (Fig 2B).

**Table 1:** MeV genome sequencing results.

	Sample	Ct MeV	Ct RNAseP	Ct 16S	Total Reads	# Mapped Reads	% Mapped Reads	% Coverage	Min Depth	Max Depth	Avg Depth
S1	Untreated	16.78	36.11	23.49	21,447,402	28,567	0.13	100	39	201	178.8
	Omnicleave	16.49	35.62	27.34	25,064,751	28,936	0.12	100	36	202	180.2
	Filter	18.12	38.19	28	26,664,762	27,352	0.10	100	29	199	170.1
S2	Untreated	21.81	36.87	20.11	26,452,172	11,916	0.05	100	13	152	73.1
	Omnicleave	21.34	38.56	17.33	44,816,272	7,123	0.02	100	4	131	43.5
	Filter	20.63	40.33	28.22	23,389,109	17,095	0.07	100	26	186	101.1
S3	Untreated	20.62	38.39	16.72	44,963,958	10,099	0.02	100	8	149	60.3
	Omnicleave	21.21	39.87	18.28	35,525,481	15,447	0.04	100	13	170	96.4
	Filter	24.51	41.26	26.82	29,117,330	4,581	0.02	100	2	115	27
S4	Untreated	27.02	36.92	31.24	28,304,111	2,103	0.01	99	1	77	11.8
	Omnicleave	26.55	38.69	30.09	22,567,411	2,439	0.01	99.8	1	72	14.2
	Filter	27.96	41.83	31.09	27,125,650	9,175	0.03	100	8	143	55.4

### Scaling sequencing resources via in-silico down-sampling

In-silico down-sampling was used to infer the minimum amount of total sequences needed to obtain complete MeV genome coverage. Each already-sequenced sample was re-analyzed, where the number of total sequences was bioinformatically changed from 15M (the number of sequences in which all samples exhibited 100% coverage, with the exception of untreated S4) to 10,000K. At each point, MeV coverage (proportion of positions sequenced out of MeV complete genome) and depth (number of repeats at each MeV genome position) were compared across the different enrichment treatments or lack thereof (S2 Table in S1 File).

**Fig 3:**
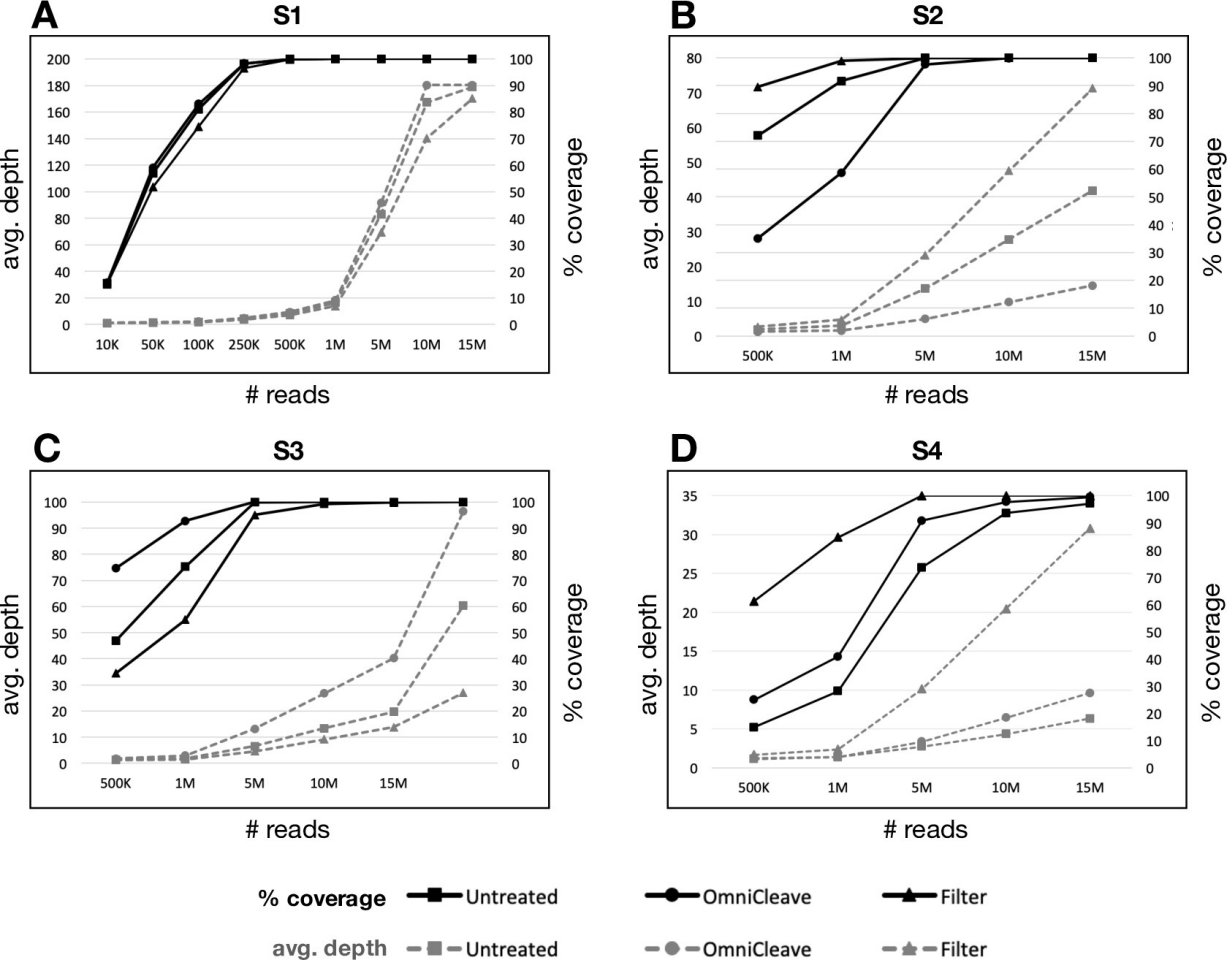
Effect of in-silico down-sampling on MeV genome coverage and depth. The total number of sequence reads were computationally down-sampled from 15M down to 10K by increments of 5M reads, for each set of samples (a- S1, b- S2, c- S3, d- S4), prior to and following OmniCleave or 0.45μm filter. % coverage: proportion of positions sequenced out of MeV complete genome; avg. depth: average number of repeats in each position of MeV complete genome.

S1 treated and untreated samples, that had high MeV quantities prior to and following treatments (Ct = 16–18, Table 1), exhibited 100% coverage even at 250K sequence reads, with an average depth of ~4 (Fig 3A). However, only with 5M reads, the minimum depth exceeded 1 or 2 (S2 Table in S1 File).

In S2 and S4, filter was advantageous compared to nuclease treatment. S2 had intermediate MeV quantities prior to enrichment (Ct~22) and filtering purified the sample from both host cell and bacterial content by orders of magnitude (Table 1) and increased the coverage and sequencing depth. S2 exhibited 99% coverage with filter treatment with 1M reads compared to 91% and 58%, respectively, in untreated and following nuclease treatment (Fig 3B). However, average depth was relatively low (<5) and the minimum depth was 1 in all samples (S2 Table in S1 File). With allocation of 5M reads, the filter-treated sample had both 100% coverage and high average depth of 23 compared to the untreated and nuclease-treated samples which had lower coverages (99.9% and 97% respectively) and lower average depths (<20, Fig 3B). Similarly, the S4 sample with low MeV quantities prior to treatments (Ct = 27), had 100% coverage at 5M sequences with an average depth of ~10 following filter treatment, while the untreated and nuclease-treated samples had coverages of 90% and 73%, respectively, and average depths of <5 (Fig 3D).

On the contrary, in sample S3 with intermediate MeV quantities prior to treatment (Ct~20), same as S2, MeV content was significantly reduced following filtering but not nuclease treatment (Table 1). With allocation of only 1M sequences, the nuclease-treated sample had 93% coverage and had higher average depth compared to the untreated (75% coverage) and filter-treated (55% coverage) samples (Fig 3C). At 5M sequences, the nuclease-treated sample reached full coverage and an average depth of >10, while the untreated and filter-treated samples did not (Fig 3C and (S2 Table in S1 File)). Based on these analyses, the amount of reads to be allocated to achieve full coverage and sufficient sequencing depth was assessed for samples with varying Ct values. To note, a degree of variation in the coverage and depth of sequencing is expected due to the clinical nature of the samples and challenges in robust MeV enrichment.

### Limited-resource sequencing achieves MeV full coverage

**Fig 4:**
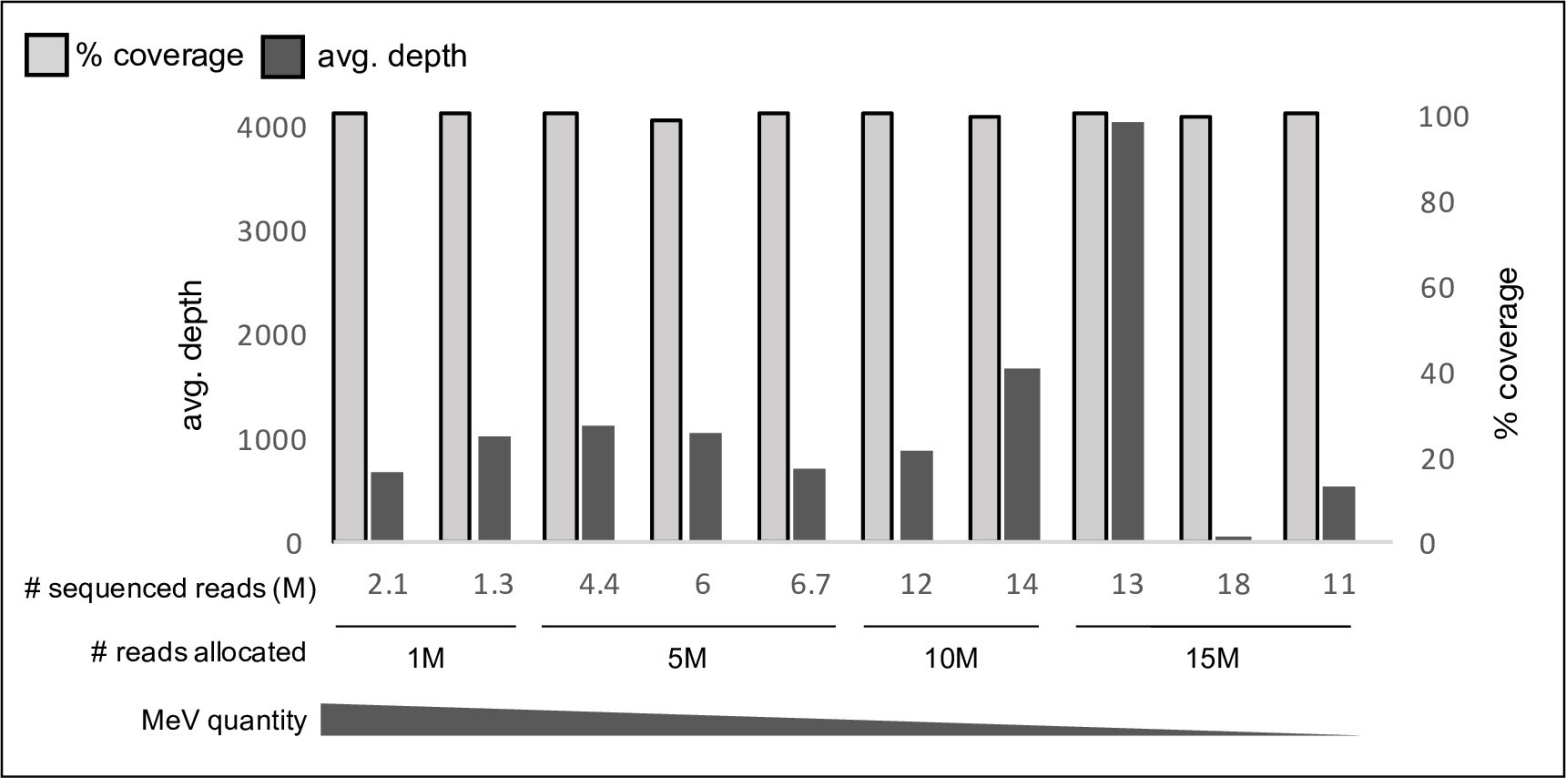
MeV genome coverage and depth following limited resource sequencing. Limited sequencing read allocation was applied to 10 MeV-positive samples following pretreatment with OmniCleave. The % coverage (right side, in grey) and average sequencing depth (left side, in black) following sequencing is shown for each sample. 1,5,10 and 15M reads were allocated (# reads allocated) according to MeV quantities in each sample, where samples with lower MeV quantities were allocated more reads and vice versa. The actual number of resulting reads per sample following sequencing was in the range of the number of allocated reads during library preparation (# sequenced reads).

Next, we examined whether limited-sequence allocation, following the guidelines inferred by in-silico down-sampling, can yield full MeV genome coverage. 10 MeV-positive samples with various MeV Ct values were treated with nuclease and sequenced with specific sequence allocation based on the MeV quantity in each sample: 1M reads for samples with Ct<20, 5M reads for samples with Ct~20, 10M reads for samples with 20<Ct<30 and 15M reads for samples with Ct>30 (S3 Table in S1 File). Sample-specific sequence allocation was applied by pooling relative quantities from each sample during library preparation (methods section). Resulting total sequences obtained following sequencing was in the range of the sequences allocated during library preparation (Fig 4, (S3 Table in S1 File), and all had >98% coverage and average sequencing depths >50 (Fig 4). Demonstrating, that complete MeV genome coverage can be achieved with limited sequence allocation, based on MeV quantity in each sample. Importantly, complete MeV coverages were obtained even for samples with low viral quantity (Ct>30), albeit using higher number of allocated sequences.

## Discussion

Measles outbreaks escalated globally in recent years and still continue to pose a threat. The growing mass of cases challenge viral surveillance and construction of reliable transmission chains. Molecular analyses utilizing MeV complete genomes enhance the resolution of such investigations, however current methods involving culturing and/or sanger amplicon-based sequencing cannot realistically be applied to the numerous samples needed for representation of large-scale outbreaks. This study assessed a practical approach for complete MeV genome sequencing, directly from patient urine samples, to be rapidly applied to numerous samples. MeV-positive urine samples that collected during the 2018–9 measles outbreak in Israel, where >4000 urine samples were processed in Israel’s national virology laboratory, were used in this study. Urine samples are more sterile and thus the virus is better retained in and for longer, compared to nasopharyngeal (NF) swabs, which are also collected to test for MeV. Also, NF swabs were mainly available from more severe hospitalized patients, as they require specialized tubes and testing techniques not always available in community clinics, and thus may represent a biased population. Both filtering and endonuclease enrichments, which are quickly and simply applied to patient urine samples, were successful in reducing background human and bacterial genomes without significantly reducing MeV quantities, and may be used interchangeably. In some cases, one treatment was favorable over the other, however as each individual urine sample may have a unique biological and chemical composition, it challenges attempts to enrich MeV signal in a robust manner. Enrichment treatments also enhanced MeV genome coverage and depth, especially in samples with intermediate and low MeV quantities, further demonstrating the advantage of enrichment. In terms of safety, application of endonuclease includes simple pipetting, however filtering involves syringe usage, thus due to potential risk of injuries, the number of samples and time constraints should be considered. To note, treatments were applied to urine samples and their application to NF samples, although likely similar, will have to be further examined. The number of sequences allocated per sample, essential in planning direct MeV sequencing from numerous samples with limited resources, was found to highly depend on MeV quantities, which may also be affected due to the heterogeneous nature of the samples, as discussed earlier. In-silico down-sampling inferred the minimum number of sequences needed to obtain complete MeV genome coverage with varying depths. To note, NGS data containing greater depths render greater confidence in making base calls, where depth and sequencer-derived errors are not uniform across the genome [15]. To obtain complete MeV genomes with ~X10 depth, down-sampling results confer the following guidelines: allocation of ~1M and ~5M sequences in samples with high (Ct<18) and intermediate (Ct~20) MeV quantities, respectively. Complete coverages are also obtainable in samples with higher MeV quantities (Ct>27), but require higher allocation of ~10M sequences. To note, higher/lower depths can be obtained given adjustments to the number of allocated sequences.

**Fig 5:**
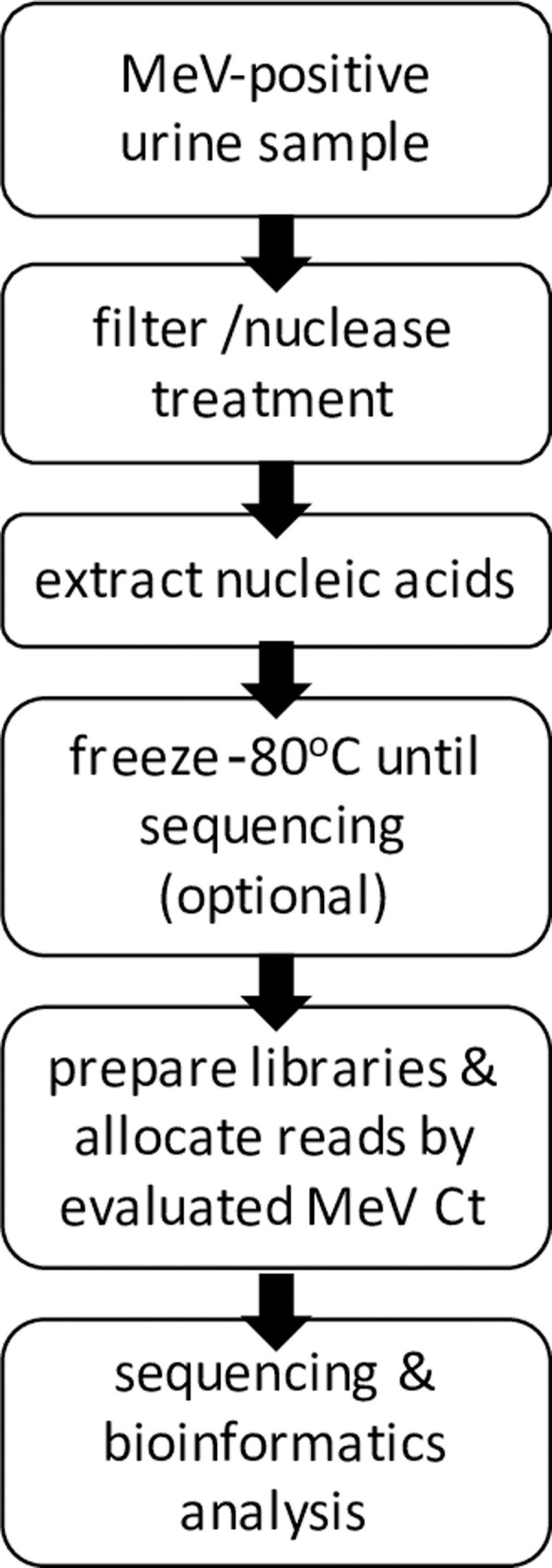
Workflow for sequencing MeV complete genomes directly from patient urine samples.

To summarize, our approach constitutes a workflow for efficient and unbiased sequencing of complete MeV genomes, which can be applied during a large-scale measles outbreak, and includes the following steps: (1) applying enrichment treatment to MeV-positive urine samples (optional for samples with Ct<18), (2) extracting nucleic acids and (optional) freezing in -80°C until sequencing, (3) preparing libraries and pooling relative quantities based on MeV Ct in each sample, (4) sequencing and analysis (Fig 5). The number of samples to be multiplexed in the same sequencing run depends on the MeV quantity in each sample. The higher the MeV quantity, the less sequences to allocate and the more samples that fit in the same sequencing run. Altogether, our study demonstrates successful sequencing of complete MeV genomes directly from patients’ urine samples, even in samples with low MeV quantities. This optimized practical approach can enable characterization of large-scale, limited-genotype measles outbreaks which are expanding globally, to enhance the resolution of epidemiological investigations and inform public health authorities, supporting measles elimination efforts, impact vaccination and outbreak preparedness policies worldwide.

## Supporting information

S1 File (DOCX)
