# Supplementary material for: Direct sequencing of measles virus complete genomes in the midst of a large-scale outbreak
Source: PLoS One. 2021 Sep 10;16(9):e0255663. doi: 10.1371/journal.pone.0255663 (PMC8432851; doi:10.1371/journal.pone.0255663)

**S1 File**

**Supplementary methods**

Real-time RT-PCR

Amplification was performed in 25µl reaction volumes, including 5µl of sample and 20µl of RT-PCR master mix. Single-use viral RNA positive control set at ~Ct=30 was used for each target. RNAseP internal control was used as previously reported [13]. RT-PCR program was: (1) 48ºc for 30:00 min, (2) 95ºc for 10:00 min, (3) 95ºc for 15 seconds 50 repeats, (4) 60ºc 1 min. Primer and probe sequences: RNAseP [14], forward: AGATTTGGACCTGCGAGCG, reverse: GAGCGGCTGTCTCCACAAGT, probe: Cy5-TTCTGACCTGAAGGCTCTGCGCG-BHQ2; MeV [15], forward: CCCTGAGGGATTCAACATGATTCT, reverse: ATCCACCTTCTTAGCTCCGAATC, probe: FAM-TCTTGCTCGCAAAGGCGGTTACGG-BHQ-1; 16S rRNA [16], forward: TGGAGC ATGTGGTTTAATTCGATGGAGCATGTGGTTTAATTCGA, reverse: TGCGGGACTTAACCCAACATGCGGGACTTAACCCAACA, probe (FAM): CA CGA GCT GAC GAC AAC CAT GCA-BHQ-CACGAGCTGACGACAACCATGCA.

**Supplementary figures and tables**

Figure S1. Ct values prior to and following enrichment treatments. (a) 16S Ct values prior to and following treatments with OmniCleave (OC) and 0.45µm filter (1F). (b) paired representation of 16S Ct values prior to and following OmniCleave (OC) treatment. Samples with high and low bacterial content prior to treatment are emphasized by the dashed line at Ct=30. OmniCleave treatment was slightly more effective for samples with Ct<30 (high bacterial content), as shown by the paired representation. (c) paired representation of 16S Ct values prior to and following 0.45µm filter (1F) treatment. Samples with high and low bacterial content prior to treatment are emphasized by the dashed line at Ct=30. 0.45µm filter (1F) treatment was significantly more effective for samples with Ct<30 (high bacterial content, median Ct values increased from 25 to 29, p-value=0.0009), compared to samples with Ct>30 (low bacterial content, median Ct values prior to and following filter treatment were 32, p-value>0.05).

Table S1. MeV, RNAseP and 16S Ct values prior to and following enrichment treatments. Sequenced samples S1-S4 are circled.


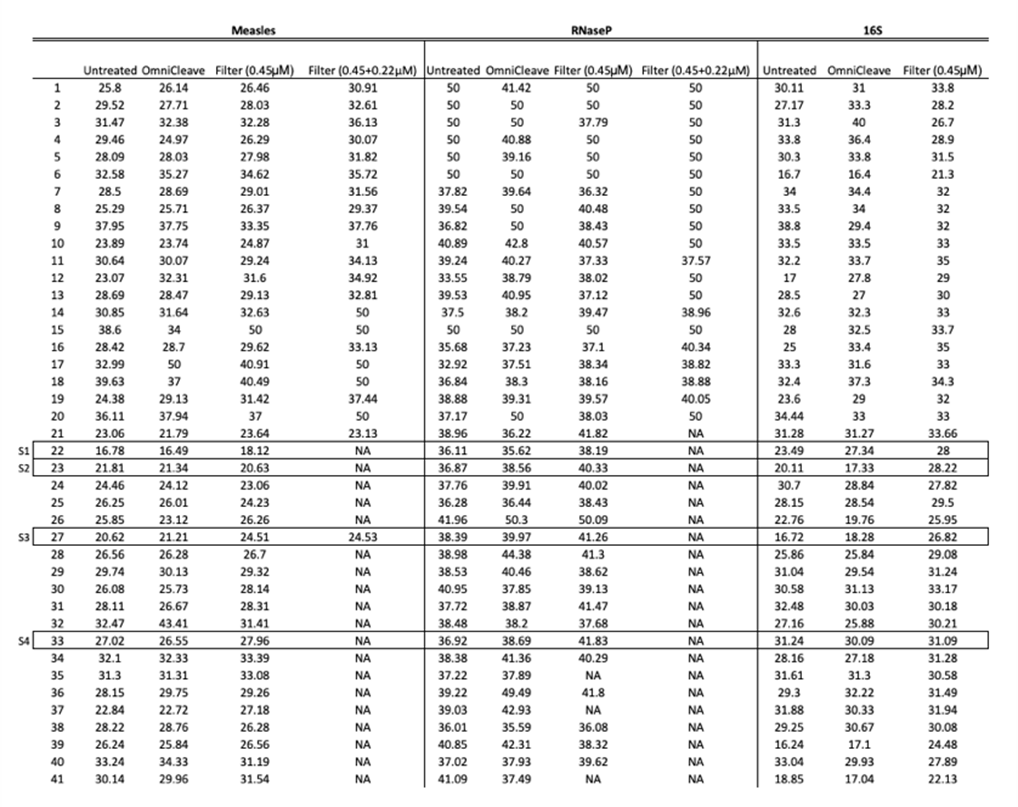


Table S2. MeV coverage and depth obtained in down-sampling


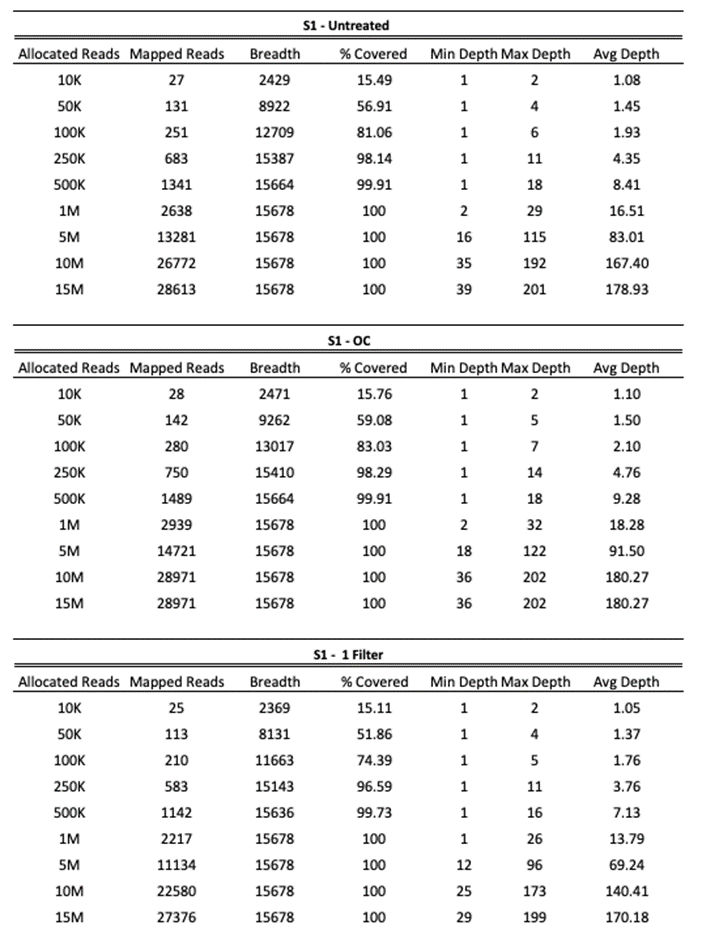


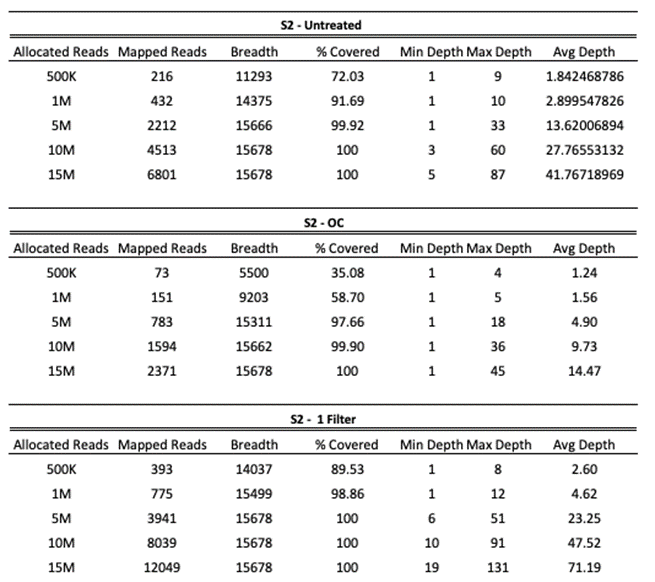


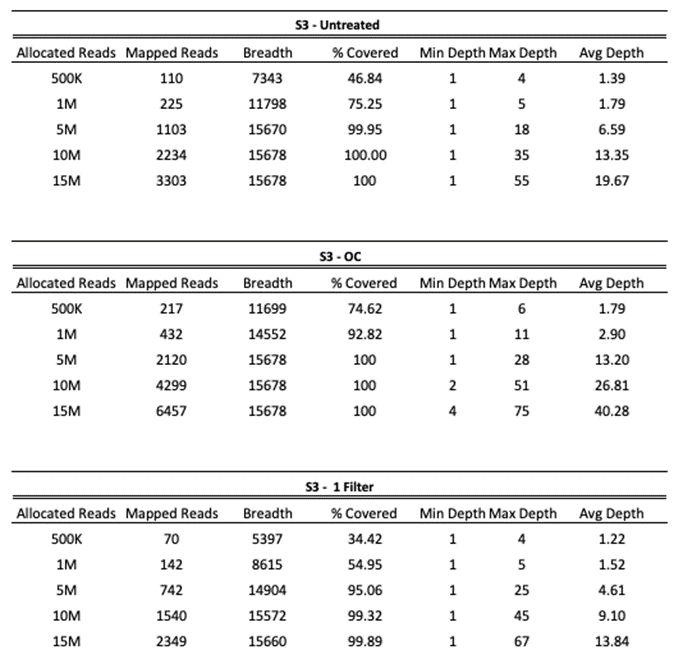


Table S3. MeV coverage and depth obtained in limited allocation sequencing


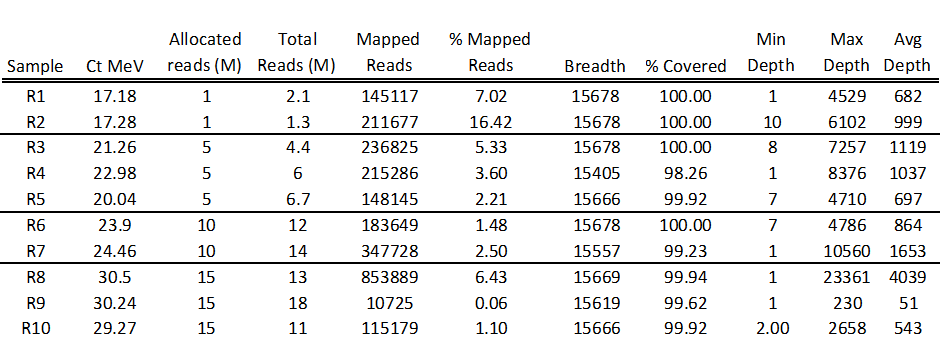

Supplement: S1 File — (DOCX) [file pone.0255663.s001.docx]
